# High prevalence of carriage of *mcr-1*-positive enteric bacteria among healthy children from rural communities in the Chaco region, Bolivia, September to October 2016

**DOI:** 10.2807/1560-7917.ES.2018.23.45.1800115

**Published:** 2018-11-08

**Authors:** Tommaso Giani, Samanta Sennati, Alberto Antonelli, Vincenzo Di Pilato, Tiziana di Maggio, Antonia Mantella, Claudia Niccolai, Michele Spinicci, Joaquín Monasterio, Paul Castellanos, Mirtha Martinez, Fausto Contreras, Dorian Balderrama Villaroel, Esther Damiani, Sdenka Maury, Rodolfo Rocabado, Lucia Pallecchi, Alessandro Bartoloni, Gian Maria Rossolini

**Affiliations:** 1 Department of Medical Biotechnologies, University of Siena, Siena, Italy; 2 Department of Experimental and Clinical Medicine, University of Florence, Florence, Italy; 3 Servicio Departamental de Salud (SEDES) de Santa Cruz, Santa Cruz, Bolivia; 4 Servicio Departamental de Salud (SEDES) de Tarija, Tarija, Bolivia; 5 Servicio Nacional de Sanidad Agropecuaria e Inocuidad Alimentaria (SENASAG), Ministerio de Desarrollo Rural y Tierras, Santa Cruz, Bolivia; 6 Instituto Nacional de Laboratorios de Salud (INLASA), Ministerio de Salud, La Paz, Bolivia; 7 Unidad Epidemiología, Ministerio de Salud, La Paz, Bolivia; 8 Servicios Generales de Salud, Ministerio de Salud, La Paz, Bolivia; 9 Infectious and Tropical Diseases Unit, Careggi University Hospital, Florence, Italy; 10 Clinical Microbiology and Virology Unit, Careggi University Hospital, Florence, Italy

**Keywords:** polymyxin, South America, Bolivia, *mcr-1*, *mcr-2*, *mcr-1.5*, rural communities, antimicrobial resistance, AMR, susceptibility testing, whole genome sequencing, WGS

## Abstract

**Background:**

The *mcr-1* gene is a transferable resistance determinant against colistin, a last-resort antimicrobial for infections caused by multi-resistant Gram-negatives.

**Aim:**

To study carriage of antibiotic-resistant bacteria in healthy school children as part of a helminth control and antimicrobial resistance survey in the Bolivian Chaco region.

**Methods:**

From September to October 2016 we collected faecal samples from healthy children in eight rural villages. Samples were screened for *mcr-1*- and *mcr-2* genes. Antimicrobial susceptibility testing was performed, and a subset of 18 isolates representative of individuals from different villages was analysed by whole genome sequencing (WGS).

**Results:**

We included 337 children (mean age: 9.2 years, range: 7–11; 53% females). The proportion of *mcr-1* carriers was high (38.3%) and present in all villages; only four children had previous antibiotic exposure. One or more *mcr-1*-positive isolates were recovered from 129 positive samples, yielding a total of 173 isolates (171 *Escherichia coli*, 1 *Citrobacter europaeus*, 1 *Enterobacter hormaechei*). No *mcr-2* was detected. Co-resistance to other antimicrobials varied in *mcr*-positive *E. coli*. All 171 isolates were susceptible to carbapenems and tigecycline; 41 (24.0%) were extended-spectrum β-lactamase producers and most of them (37/41) carried *bla*_CTX-M_-type genes. WGS revealed heterogeneity of clonal lineages and *mcr*-genetic supports.

**Conclusion:**

This high prevalence of *mcr-1*-like carriage, in absence of professional exposure, is unexpected. Its extent at the national level should be investigated with priority. Possible causes should be studied; they may include unrestricted use of colistin in veterinary medicine and animal breeding, and importation of *mcr-1*-positive bacteria via food and animals.

## Background

The *mcr-1* gene is a transferable colistin resistance determinant that was first described among enterobacterial strains isolated from animals and humans in China. The gene encodes a phosphoethanolamine transferase that modifies the colistin target by addition of phosphoethanolamine to the 1’ or 4’ phosphate group of lipid A, which reduces its affinity to colistin [1,2]. Discovery of *mcr-1* was considered highly alarming, given the role that colistin has recently regained as a last-resort antibiotic for treatment of infections caused by multi-resistant Gram-negative pathogens such as carbapenem-resistant Enterobacterales and *Acinetobacter baumannii* [1,3].

Subsequent to its discovery, several studies have revealed a global distribution of *mcr-1*, with an overall higher prevalence among *Escherichia coli* and *Salmonella enterica*, and occasional occurrence in other enterobacterial species. Most *mcr-1*-positive strains were of animal origin, and farm animals were identified as the principal reservoir of *mcr-1* genes [1,4]. Investigation of archival strains dated the presence of *mcr-1* back to at least the 1980s [5]. As with other resistance genes, minor allelic variants of *mcr-1* have been detected [6]. More recently, additional transferable *mcr* genes (*mcr-2*, *mcr-3*, *mcr-4*, *mcr-5, mcr-6*, *mcr-7* and *mcr-8*) have been reported, for which the global epidemiology remains to be clarified [7-13].

In South America, *mcr-1* genes have been reported from several countries in isolates from humans, animals and food [14-27]. Recently, the Pan American Health Organisation (PAHO) section of the World Health Organization (WHO) recommended to implement and strengthen surveillance and epidemiological investigation of plasmid-mediated transferable colistin resistance in its Member States [14]. In Bolivia, *mcr-1* has thus far been reported in a *Citrobacter braakii* that was isolated from a ready-to-eat food sample [21], as well as in a few clinical isolates of *E. coli* referred from various departments to the National Institute of Health Laboratories (INLASA) (data not shown).

During the last two decades we carried out several surveillance studies in the Bolivian Chaco region, documenting a high prevalence of resistance to old and more recent antibiotics in commensal and pathogenic bacteria from humans [21,28-32].

In 2016, a new surveillance study was carried out in a population of healthy school children from several rural communities in this region to investigate the prevalence of intestinal parasites and the carriage of antibiotic-resistant bacteria. Here we report about an unexpected and high rate of faecal carriage of *mcr-1*-positive Enterobacterales in this population.

## Methods

### Study population and setting

**Figure 1:**
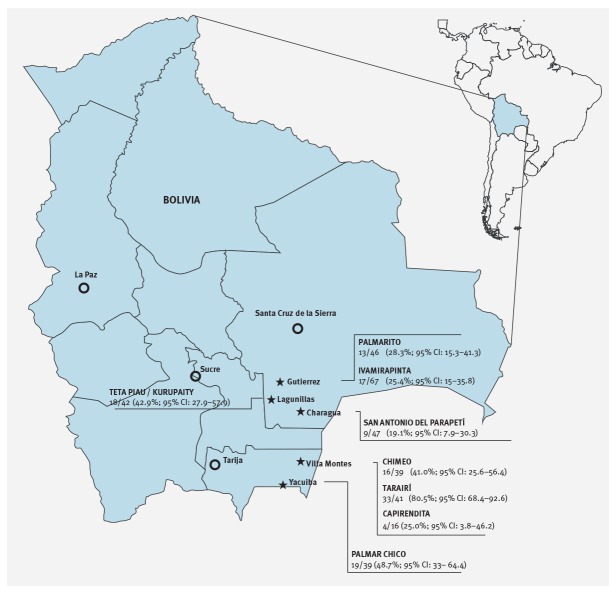
Geographical locations of the surveyed communities and the proportion of *mcr-1*-positive samples, Chaco, Boliva, September–October 2016 (n = 8 communities)

The study population consisted of healthy school children living in eight rural communities of the Chaco region, in south-eastern Bolivia (between longitude 63°66 and 63°18 east and latitude 19°49 and 21°88 south, Figure 1). In these communities, the population lives in houses mostly constructed of mud and sticks, with packed earth floors and straw or corrugated metal roofs. There is no wired electricity and no sewage system. The main water sources are small ponds, in which animals also bathe and drink, and outdoor taps. The economy is mostly based on subsistence farming and local animal husbandry.

In each community, children were selected among those attending primary school, starting from the third year and possibly including the upper years, to achieve a number of ca 50 individuals per site whenever possible. This sample size corresponded to that recommended by WHO for cluster sampling in helminth control programmes in healthy school children [33].

Previous use of antibiotics during the last 15 days was investigated by a questionnaire administered to parents.

### Laboratory analyses

#### Screening for *mcr-1*- and *mcr-2*-positive strains in faecal samples

One faecal sample for each child was collected during a two-month period from September to October 2016; the samples were transferred to the Laboratories of Camiri or Villa Montes Hospitals within 6 hours and were plated onto MacConkey agar. After incubation at 35 °C for 24 hours, the bacterial growth (representative of the total enterobacterial microbiota) was collected with a sterile swab in an Amies transport medium and was shipped to Italy. Each sample was then subcultured on MacConkey agar again, and the bacterial growth was resuspended in Brain Heart Infusion broth plus 20% (v/v) glycerol and stored at -70 °C pending further analyses.

To screen for the presence of *mcr-1*- and *mcr-2*-positive strains, the preserved suspensions of total enterobacterial microbiota were thawed and 10 μl were inoculated onto McConkey supplemented with colistin (2 mg/L, MCC medium). After incubation at 35 °C for 24 hours, a loopful of the bacterial growth (taken either from confluent growth or from isolated colonies of different morphologies) was resuspended in 300μl of normal saline, and half of the bacterial suspension was used to prepare a crude DNA extract by heating at 99 °C for 15 minutes. The crude extracts were then screened for the presence of *mcr-1* and *mcr-2* genes by real-time (RT) PCR, as described previously [34]. In the case of a positive result, the remaining bacterial suspension was used to inoculate the MCC medium to obtain isolated colonies, and all isolated colonies of different morphology were then tested for the presence of *mcr* genes by RT-PCR. The *mcr-*positive isolates were identified using MALDI-TOF mass spectrometry (Vitek MS, bioMérieux, Marcy-l’Etoile, France).

When a sample yielded two or more *mcr-1*-positive isolates of the same species, clonal relatedness of the isolates was investigated by random amplification of polymorphic DNA (RAPD) profiling, as described previously [35]. The three *mcr*-positive isolates that were colistin susceptible were subjected to *mcr* gene amplification and sequencing using previously described primers and conditions [34].

#### Antimicrobial susceptibility testing

Antimicrobial susceptibility testing was carried out using reference broth microdilution [36]. Minimum inhibitory concentration (MIC) results were interpreted according to the European Committee on Antimicrobial Susceptibility Testing (EUCAST) clinical breakpoints [36].

#### Analysis of extended-spectrum β-lactamases

All isolates showing a ceftazidime and/or cefotaxime MIC > 1 mg/L were screened for extended-spectrum β-lactamases (ESBL) production by a combination disk test using ceftazidime and cefotaxime as substrates and clavulanic acid as an inhibitor [37]. ESBL-positive isolates by phenotypic testing were subjected to RT-PCR for the detection of *bla*_CTX-M_ ESBL genes, as described previously [38].

#### Whole genome sequencing

A subset of 18 *mcr-1*-positive isolates were subjected to whole genome sequencing (WGS) analysis. This subset comprised two *E. coli* isolates per community: from randomly selected individuals co-colonised by two different *mcr-1*-positive *E. coli* or from two randomly selected individuals if co-colonisations were not detected and the two non-*E. coli* isolates bore *mcr-1*. For the latter, species identification was carried out by the analysis of housekeeping genes [39,40]. Bacterial genomic DNA of these 18 selected *mcr*-positive isolates, extracted using the phenol-chloroform method [41], was subjected to WGS with a MiSeq platform (Illumina, Inc., San Diego, California, United States (US)) using a 2x300 paired-end approach. Raw reads were assembled using SPAdes 3.5 [42]. An average of 120 contigs per strain was obtained, with an average N50 of 163 Kb. Draft genomes have been deposited in the National Center for Biotechnology Information (NCBI) WGS database under the BioProject PRJNA427943 (accession numbers: PQTO00000000; PQTN00000000; PQTM00000000; PQTL00000000; PQTK00000000; PQTJ00000000; PQTI00000000; PQTH00000000; PQTG00000000; PQTF00000000; PQTE00000000; PQTD00000000; PQTC00000000; PQTB00000000; PQTA00000000; PQSZ00000000; PQSY00000000; PQSX00000000). Resistance genes and plasmid content were investigated using the ResFinder and PlasmidFinder tools available at the Center for Genomic Epidemiology at https://cge.cbs.dtu.dk/services/ResFinder/. Clonal relatedness was investigated by in silico determination of the multilocus sequence typing (MLST) profile obtained by the MLST 1.8 software (available at https://cge.cbs.dtu.dk/services/MLST/) using the assembled WGS as input data.

### Statistical analysis

Statistical analysis of the data was performed with STATA 11.0 (StataCorp, College Statio, Texas, US). Frequencies and percentages with 95% confidence intervals (CI) for categorical variables, medians and interquartile ranges (IQR) for continuous variables were calculated. Mann–Whitney test was used to compare median age. Chi-squared test was used to investigate the association of *mcr-1* carriage with sex and prior antibiotic use. Results were considered significant when the p value was ≤ 0.05.

### Ethical statement

Written informed consent was always obtained from parents or legal guardians. The investigation was planned and carried out within a collaboration agreement between the Ministry of Health of the Plurinational State of Bolivia and the University of Florence, Italy, and with the support of the Guaraní political organisation (Asamblea del Pueblo Guaraní). Ethical approval for the study was obtained from the above-mentioned institutions (see Acknowledgements section).

## Results

Faecal specimens were obtained from 337 healthy school children in eight rural communities of the Bolivian Chaco region (Figure 1). Children (179 females; 53%;) were aged 7 to 11 years (mean: 9.2 years). Previous antibiotic exposure was only reported for four children.

CI: confidence interval.

The surveyed communities are located in the Bolivian Chaco, in rural areas of five municipalities (indicated with stars). For each community, the proportion of *mcr-1*-positive samples versus the total number of collected samples is reported, along with 95% CI. Major Bolivian cities are indicated by circles.

### *mcr-1* carriage

All 337 samples of enterobacterial microbiota yielded some growth (from scanty to vigorous) on the MCC medium, and 129 (38.3%) yielded a positive result for *mcr-1*. Positive samples were detected in children from each village, although at variable rates (range: 19.1–80.5%; Figure 1). No *mcr-2* genes were detected. One or more *mcr-1*-positive isolates were recovered from each of the 129 samples, yielding a total of 173 positive isolates, including 171 *E. coli*, one *Citrobacter* spp. and one *Enterobacter* spp.. Multiple *mcr*-*1*-positive isolates from the same sample consisted of either two or three *E. coli* isolates of different colonial morphology and RAPD profile (in 32 and 5 samples, respectively), or in an *E. coli* plus an *Enterobacter* spp. (in one sample).

No differences were found in the demographic characteristics, sex or age, of children carrying *mcr-1*-positive Enterobacterales or children without *mcr-1* carriage (Table 1), nor were there any differences in the living conditions of the communities with different proportions of carriers (data not shown).

**Table 1:** Features of the study population, stratified by *mcr*-1-positive and -negative children, Chaco, Bolivia, September–October 2016

Characteristics	Total (n/N)	%	*mcr-1*-negative (n/N)	%	*mcr-1*-positive (n/N)	%	P value
**Sex**							
Male	158/337	47	100/208	48	58/129	45	0.58
Female	179/337	53	108/208	52	71/129	55	
**Age (years)**							
Mean (95% CI)	9.3 (9.1–9.4)	NA	9.3 (9.1–9.5)	NA	9.2 (9.0–9.5)	NA	0.81
Median (IQR)	9 (8–10)	NA	9 (8–10)	NA	9 (8–10)	NA	
**Prior antibiotic use^a^**	4/337	1	3/208	1	1/129	1	0.58

CI: confidence interval; IQR: interquartile range; NA: not applicable.

^a^In the last 15 days.

### Antimicrobial susceptibility of *mcr-1*-positive isolates

Colistin susceptibility testing showed that the majority (n = 170; 98.3%) of the *mcr-1*-positive isolates were resistant to colistin (MIC range: 4–> 8 mg/L), while only three *E. coli* (from different villages) were colistin-susceptible (all with an MIC of 2 mg/L) (Table 2). Sequencing of *mcr* amplicons from the latter isolates showed identity with *mcr-1*, suggesting that the colistin susceptible phenotype was not due to mutations inactivating the gene. Variable resistance rates to other antimicrobial agents were observed, including fluoroquinolones, expanded-spectrum cephalosporins, β-lactamase plus inhibitor combinations and gentamicin. All isolates were susceptible to carbapenems and tigecycline (Table 2).

**Table 2:** Susceptibility of *mcr 1*-positive *Escherichia coli* isolates to various antimicrobials, Chaco, Bolivia, September–October 2016 (n = 171)

AMC		PTZ		CAZ		CTX		FEP		MEM		ERT		GEN		CIP		TIG		COL		Total	
n	%	n	%	n	%	n	%	n	%	n	%	n	%	n	%	n	%	n	%	n	%	n	%
72	42.1	171	100	130	76.0	128	74.9	130	76.0	171	100	171	100	138	80.7	62	36.3	171	100	168	1.7	171	100

AMC: amoxicillin/clavulanate (clavulanate at fixed concentration of 4 mg/L); CAZ: ceftazidime; CIP: ciprofloxacin; COL: colistin; CTX: cefotaxime; ERT: ertapenem; FEP: cefepime; GEN: gentamicin; MEM: meropenem; PTZ: piperacillin/tazobactam (tazobactam at fixed concentration of 4 mg/L); TIG: tigecycline.

Numbers and percentages of susceptible isolates are given.

All 41 isolates showing a ceftazidime and/or cefotaxime MIC > 1 mg/L were positive for ESBL production by combination disk synergy test, and 37 of them were positive for the presence of a *bla*_CTX-M_-type ESBL gene.

### Diversity of the *mcr-1-*positive isolates

WGS analysis of the subset of 18 *mcr*-*1*-positive isolates confirmed the identification of the two non-*E. coli* isolates as *Citrobacter europaeus* and *Enterobacter hormaechei*, respectively (Table 3), two species in which *mcr-1* was not previously reported.

**Table 3:** Features of *mcr-1*-positive isolates subjected to whole genome sequencing analysis, Chaco, Bolivia, September–October 2016 (n = 18)

Community	Isolate code	Subject code	Species	Additional resistance trait(s)^a^	Acquired resistance genes^b^	ST^c^	*mcr* variant and genetic context^d^	*mcr* contig size (bp)
Palmarito	12A	1	*Escherichia coli*	*AMC; GEN; CIP*	*blaTEM-1B; aac (3)-IV; aph (4)-Ia; fosA3; floR; qnrB19; tet(A)*	48	*mcr-1*-*pap* (IncI2)	61,600
	12B	1	*E. coli*	*GEN; CIP*	*blaTEM-1B; aac (3)-IV; aph(3’)-Ia; aph (4)-Ia; strA; strB; catA1; floR; oqxA; oqxB; sul2; tet(A)*	744	*mcr-1*-*pap* (IncI2)	60,992
Ivamirapinta	155A	2	*E. coli*	FS^e^	ND	10	*mcr-1*-*pap* (IncI2)	60,547
	155B	2	*E. coli*	*AMC; CIP*	*blaTEM-1A; aadA1; aadA2; strA; strB; cmlA1; floR; qnrB19; sul2; sul3; tet(A); dfrA8*	206	*mcr-1*-unk^f^	2,943
Tetapiau/Kurupaity	86A	3	*E. coli*	*AMC; CIP*	*blaTEM-1B; strA; strB; floR; qnrB19; sul2; tet(A); dfrA1*	2,705	*mcr-1*-unk^f^	2,942
	86B	3	*E. coli*	*AMC; CIP*	*blaTEM-1B; aadA1; aadA2; strA; strB; cmlA1; floR; sul1; sul2; sul3; tet(A); tet(B); dfrA1; dfrA12*	2,936	*mcr-1.5-pap-*IS*Apl1* (IncHI1)	13,7897
	67A	4	*Citrobacter europaeus*	*AMC*	*qnrB19; qnrB28*	NA	*mcr-1*-*pap* (IncI2)	60,321
San Antonio del ParapetÍ	173A	5	*E. coli*	*AMC; CIP; GEN; CAZ; CTX; FEP; (ESBL)*	*blaCTX-M-55; aadA1; aadA2; aadA5; rmtB; fosA3; cmlA1; floR; qnrB19; sul3; tet(A); dfrA17*	1,286	*mcr-1*-unk^f^	6,134
	173B	5	*E. coli*	AMC; CIP; GEN; CAZ; CTX; FEP; (ESBL)	blaCTX-M-55; blaTEM-1B; aadA1; aadA2; rmtB; cmlA1; floR; qnrB19; sul3; tet(A)	1,286	*mcr-1*-unk^f^	2,863
TarairÍ	224A	6	*E. coli*	AMC	aadA1; aadA2; strA; strB; cmlA1; floR; qnrB19; sul2; sul3; tet(A); tet(B); dfrA14	2,705	*mcr-1*-*pap* (IncI2)	59,561
	224B	6	*E. coli*	AMC; CIP	blaTEM-1B; aadA1; aadA2; strA; strB; cmlA1; floR; QnrB19; sul2; sul3; tet(A); tet(B); dfrA14	7,570	∆IS*Apl1-mcr-1-pap-*IS*Apl1* (IncHI1)	52,737
Palmar Chico	306A	7	*E. coli*	AMC	blaTEM-1B; aadA5; strA; strB; sul1; sul2; dfrA17	69	*mcr-1*-*pap* (IncI2)	63,921
	306B	7	*E. coli*	AMC; CIP	blaTEM-1B; blaOXA-1; aadA1; sul1; tet(X)	10	*mcr-1*-*pap* (IncI2)	64,425
	301B	8	*Enterobacter hormaechei*	FS^e^	ND	-	*mcr-1*-*pap* (IncI2)	63,943
Capirendita	286A	9	*E. coli*	AMC	blaTEM-1B; aadA1; floR; sul3; tet(A); tet(C); dfrA1	117	*mcr-1*-*pap* (IncI2)	59,748
	295B	10	*E. coli*	FS^e^	ND	711	*mcr-1*-*pap* (IncI2)	56,317
Chimeo	274A	11	*E. coli*	AMC; GEN; CIP	blaTEM-1B; aac (3)-IV; aadA1; aadA2; aph(3’)-Ia; cmlA1; floR; qnrB19; sul2; sul3; tet(A); tet(M); dfrA12	7,571	*mcr-1*-unk^f^	2,943
	274B	11	*E. coli*	AMC; CAZ; CTX; FEP (ESBL)	blaCTX-M-55; blaTEM-1B; blaOXA-10; aac(6')Ib-cr; aacA4; aadA1; strA; strB; fosA3; cmlA1; floR; qnrB19; qnrVC4; sul2; tet(A); dfrA14	3,056	*mcr-1*-*pap* (IncI2)	60,652

AMC: amoxicillin/clavulanate (clavulanate at fixed concentration of 4 mg/L); CAZ: ceftazidime; CIP: ciprofloxacin; COL: colistin; CTX: cefotaxime; ESBL: extended-spectrum β-lactamase; FEP: cefepime; FS: fully susceptible; GEN: gentamicin; NA: not applicable; ND: none detected; ST: sequence type; unk: unknown.

^a^All isolates were resistant to colistin; additional resistance traits referred to the panel of tested drugs reported in Table 2.

^b^Acquired resistance genes as determined by analysis with the ResFinder software.

^c^Sequence-types were assigned using the Warwick scheme (http://enterobase.warwick.ac.uk/species/index/ecoli).

^d^If the gene was linked with a known plasmid backbone, the plasmid replicon type is reported in brackets.

^e^The isolate was susceptible to all tested agents except colistin.

^f^In these cases it was not possible to reveal the nature of flanking regions due to the presence of repeated sequences flanking the gene.

For each isolate, the epidemiological data, additional resistance profile, acquired resistance genes content, sequence type and *mcr* genetic context are reported.

In silico MLST analysis of the 16 *E. coli* isolates revealed a considerable diversity, with only a few isolates from different villages belonging to the same sequence type (ST). All but one of the couples isolated from the same individual belonged to different STs (Table 3).

Analysis of the acquired resistance genes showed a remarkable diversity and a variety of patterns (Table 3). The number of known acquired resistance genes varied from 0 to 16 (median: 9). Overall, the resistance gene content was consistent with the susceptibility profile. The three ESBL-positive *E. coli* isolates carried the *bla*_CTX-M-55_ variant previously reported in Bolivia [30].

Analysis of the *mcr-1* carrying contigs revealed that in 13 isolates the *mcr-1* gene was linked to backbone regions typical of IncI2 or IncHI1 plasmids, suggesting a plasmid location, with some plasmid diversity. In the remaining five isolates, it was not possible to determine the nature of flanking regions due to the presence of repeated sequences flanking the gene (Table 3).

## Discussion

Our study revealed a very high prevalence of carriage of *mcr-1-*positive strains among healthy children living in rural communities of the Bolivian Chaco. Carriage of *mcr-1*-positive strains in healthy humans has been investigated in a limited number of studies, mostly from Asian countries [41-53]. The prevalence rates detected in such studies have usually been low (< 5%), except in a group of chicken farmers from Vietnam, where a 34.7% carriage rate of *mcr-1-*positive *E. coli* was detected and attributed to professional exposure to *mcr-1-*positive animals [45]. Therefore, to our best knowledge, we present the highest rate of *mcr-1* carriage thus far reported in healthy humans.

In our study, professional exposure could be excluded as a reason for the high prevalence of *mcr-1* carriage, as well as human use of colistin. Overall, only four children had prior exposure to antibiotics and the use of colistin in Bolivia is occasional and limited to infections by some multi-drug resistant pathogens in large urban hospitals (data not shown). However, colistin is available with no restrictions for veterinary use and in animal breeding [54], and we hypothesise that this could have played a major role in the selection of colistin-resistant strains in the animal population and the environment. Moreover, the introduction of *mcr*-positive strains via imported food and/or food-producing animals from countries where their prevalence was found to be high (e.g. Brazil) [15,22] could also represent a source of such strains. Poor sanitation and close contact with animals, which characterise the studied setting, may lead to a high level of environmental contamination and facilitate cross-transmission of colistin-resistant strains and colistin resistance genes between different environments, resulting in a high prevalence in humans who are not directly exposed to the drug.

In our case, only a minority of the *mcr*-positive isolates showed resistance to other antimicrobials, and no carbapenem resistance was detected, leaving a number of therapeutic options in case of infection. However, the potential risk of spread of the *mcr-1* gene to extensively resistant isolates through transferable plasmids mechanisms should not be underestimated.

Genomic analysis of a subset of the *mcr-1*-positive *E. coli* isolates, representative of different communities and of different isolates from the same child, revealed a remarkable heterogeneity in terms of clonal lineages and genetic supports. Therefore, the observed epidemiological scenario could not be ascribed to the expansion of a single *mcr-1*-positive clone, nor even to the spread of a single plasmid. The diversity of the genetic background of the *mcr-1* genes underlined the ability of this gene to transfer itself among different clones (and even different species) and plasmids. Interestingly, we detected for the second time in South America the *mcr-1.5* variant, previously described in an *E. coli* strain from Argentina [23].

Our study has some limitations. First, the presence of animal or environmental reservoirs of *mcr*-positive isolates and the direct transmission between humans and animals/environment could not be demonstrated, since we did not collect any samples from animals or the environment. Second, apart from *mcr-2*, we did not search for other recently described *mcr*-variants that could be responsible for resistance observed in other isolates. Third, the study was designed as a cross-sectional survey, in which one sample from each individual was collected. It would be interesting to investigate the prevalence of *mcr-1* carriage in adults and the duration of carriage over time to understand if and how much humans could represent a major reservoir in this setting. It would also be interesting to further characterise, in more detail, the plasmid supports of the *mcr-1* and other resistant determinants. Investigations on these aspects are underway.

In conclusion, our findings prompt the need to rapidly monitor the extent of human and animal carriage rates and environmental contamination by *mcr* genes with a one-health approach, and to introduce policies banning the non-therapeutic use of colistin. This was also recently highlighted by the PAHO/WHO, which encouraged the implementation of animal-human surveillance, as well as actions to prevent and control the spread of *mcr*-positive microorganisms, such as the monitoring of colistin use in human food production [14]. In Europe, knowledge of *mcr* carriage among healthy individuals is still limited [47,53]. While available data suggest a very low occurrence, it will be interesting to study human and animal carriage rates and environmental contamination in different countries and settings.
